# Is Eligibility Enough? Pretreatment Prediction of Failure to Reach a Second Cycle of [^177^Lu]Lu-PSMA Radioligand Therapy

**DOI:** 10.3390/diagnostics16203359

**Published:** 2026-10-15

**Authors:** Halim Özçevik, Enes Mustafa Kaya, Burcu Esen Akkaş, Gündüzalp Buğrahan Babacan, Ayşe Betül Dere, Berke Güner, Hatice Pekediş, Cengiz Yağmur, Meryem Kaya

**Affiliations:** 1 Ministry of Health, 06800 Ankara, Türkiye; 2 Department of Nuclear Medicine, Başakşehir Çam and Sakura City Hospital, 34480 Istanbul, Türkiye; enesmustafakaya95@gmail.com (E.M.K.); burcuesen@yahoo.com (B.E.A.); gunduzalp.b.babacan@gmail.com (G.B.B.); aysebetuldere@gmail.com (A.B.D.); berkeguner96@gmail.com (B.G.); pekedishatice@gmail.com (H.P.); cengizyagmur69@gmail.com (C.Y.); bmeryemkaya@yahoo.com (M.K.)

**Keywords:** prostate cancer, PSMA, lutetium-177, radioligand therapy, risk score, treatment attrition

## Abstract

**Background/Objectives**: Current guidelines define no absolute contraindication to prostate-specific membrane antigen (PSMA)-targeted radioligand therapy (RLT), yet some patients receive only a single cycle. We aimed to characterize this single-cycle attrition (SCA) and derive an exploratory pretreatment risk score predicting it. **Methods**: Seventy consecutive patients with metastatic castration-resistant prostate cancer treated with [^177^Lu]Lu-PSMA RLT at one center (2022–2025) were reviewed; 58 were assessable. SCA was defined as permanent non-initiation of a second cycle. The analyses employed false-discovery-rate-adjusted nonparametric comparisons, receiver operating characteristic (ROC), Kaplan–Meier and univariable Cox methods, with optimism-corrected bootstrap internal validation. **Results**: SCA occurred in 12 of 58 patients (20.7%), owing to death or rapid clinical deterioration (*n* = 8) or grade 3–4 hematotoxicity (*n* = 4); all 12 died (median overall survival 3 months). Higher C-reactive protein (CRP), lower lymphocytes and hemoglobin, and higher alkaline phosphatase (ALP) and prostate-specific antigen (PSA) separated the groups (all *q* < 0.05). The five-item Single-Cycle Attrition Risk (SCAR-5) score (one point each: CRP ≥ 16.5 mg/L, lymphocytes ≤ 1.39 × 10^9^/L, hemoglobin ≤ 9.6 g/dL, ALP ≥ 191 U/L, PSA ≥ 34.2 ng/mL) discriminated SCA with an optimism-corrected area under the ROC curve (AUC) of 0.88 (apparent AUC 0.95, 95% confidence interval (CI) 0.89–0.99). No SCA occurred below score 3 (0/39; upper 95% confidence limit 9.0%); a score ≥ 3 identified all events (sensitivity 100%, 95% CI 74–100; specificity 85%, 95% CI 71–94) and was associated with shorter survival (univariable Cox hazard ratio 3.94, 95% CI 2.01–7.74). **Conclusions**: One in five patients who initiated RLT did not proceed beyond the first cycle. SCAR-5, an exploratory score derived from routine pretreatment laboratory values, stratified risk of early treatment attrition; external prospective validation is required before clinical use.

## 1. Introduction

Radioligand therapy (RLT) targeting the prostate-specific membrane antigen (PSMA) with lutetium-177 has become an established treatment for metastatic castration-resistant prostate cancer (mCRPC). The phase 3 VISION trial demonstrated prolonged imaging-based progression-free and overall survival when [^177^Lu]Lu-PSMA-617 was added to standard of care in progressive PSMA-positive disease [1], the randomized TheraP trial reported higher prostate-specific antigen (PSA) response rates than cabazitaxel [2], and earlier prospective series had established the activity and tolerability of the approach [3]. The PSMAfore trial recently extended this evidence to taxane-naive patients [4,5]. Lu-PSMA RLT is therefore embedded in international guidelines and is delivered at rapidly growing volumes worldwide [6,7].

Patient selection for RLT nevertheless remains imperfect. The joint European Association of Nuclear Medicine/Society of Nuclear Medicine and Molecular Imaging (EANM/SNMMI) procedure guideline defines no absolute contraindication: eligibility rests on PSMA-avid disease at positron emission tomography (PET), whereas an anticipated life expectancy below 3 months, an Eastern Cooperative Oncology Group (ECOG) performance status of 3 or higher and relevant myelosuppression constitute relative contraindications requiring individual risk–benefit assessment [6]. Prognostic nomograms [8] and single-center predictor analyses [9,10] stratify expected survival, but none of these tools addresses the operational question faced at the first cycle: whether the patient will survive long enough, and tolerate treatment well enough, to receive the multi-cycle course on which the benefit demonstrated in the trials was based.

The question is clinically important because RLT delivers its benefit over multiple cycles: in VISION, benefit accrued over a median of five administrations [1], and dosimetry, response and retreatment data likewise presuppose a multi-cycle course [11]. A patient who receives a single cycle and then dies or becomes permanently treatment-intolerant has been exposed to irradiation, repeated hospital attendance and the consumption of a limited therapeutic window in end-stage disease, while the clinical benefit of that single administration remains undetermined. Such exposure is not rare: in an early tertiary-center series, 19% of patients received only one cycle [12], and early discontinuation within the first three to four cycles has been linked to adverse baseline features [13]. A recent cost-effectiveness analysis further underlined the importance of patient selection, as [^177^Lu]Lu-PSMA-617 was not cost-effective relative to cabazitaxel under base-case assumptions [14]. This failure to reach a second cycle, which we term single-cycle attrition (SCA), has nevertheless not been studied as a distinct endpoint. In routine practice, the decision to initiate RLT is made at a single time point with the information available before the first cycle; a simple instrument based on parameters already collected during eligibility work-up could therefore support the multidisciplinary discussion at that moment in everyday clinical settings.

We therefore analyzed a consecutive single-center cohort treated with Lu-PSMA RLT to quantify the incidence and causes of SCA, to compare pre-specified baseline characteristics between single-cycle and multi-cycle patients, and to derive a pretreatment risk score for this event.

## 2. Materials and Methods

### 2.1. Study Design and Patients

This retrospective cohort study included 70 consecutive patients with progressive mCRPC treated with Lu-PSMA RLT at Başakşehir Çam and Sakura City Hospital, Istanbul, Türkiye, between May 2022 and December 2025. The data cut-off was January 2026; the last included first cycle was administered in October 2025, so that every patient had a minimum potential follow-up of 3 months. Eligibility followed institutional practice consistent with the EANM/SNMMI procedure guideline [6]: PSMA-avid disease on [^68^Ga]Ga-PSMA-11 PET/computed tomography (CT), adequate organ function and multidisciplinary tumor-board approval. Treatment consisted of intravenous [^177^Lu]Lu-PSMA-617 at an activity of 7.4 GBq per cycle, administered at 6–8-week intervals, with continuation decided before each cycle on clinical, laboratory and imaging grounds. The study was conducted in accordance with the Declaration of Helsinki and approved by the institutional ethics committee (decision no. E-96317027-514.10-305880289, 24 February 2026).

### 2.2. Study Population and Exclusions

**Figure 1:**
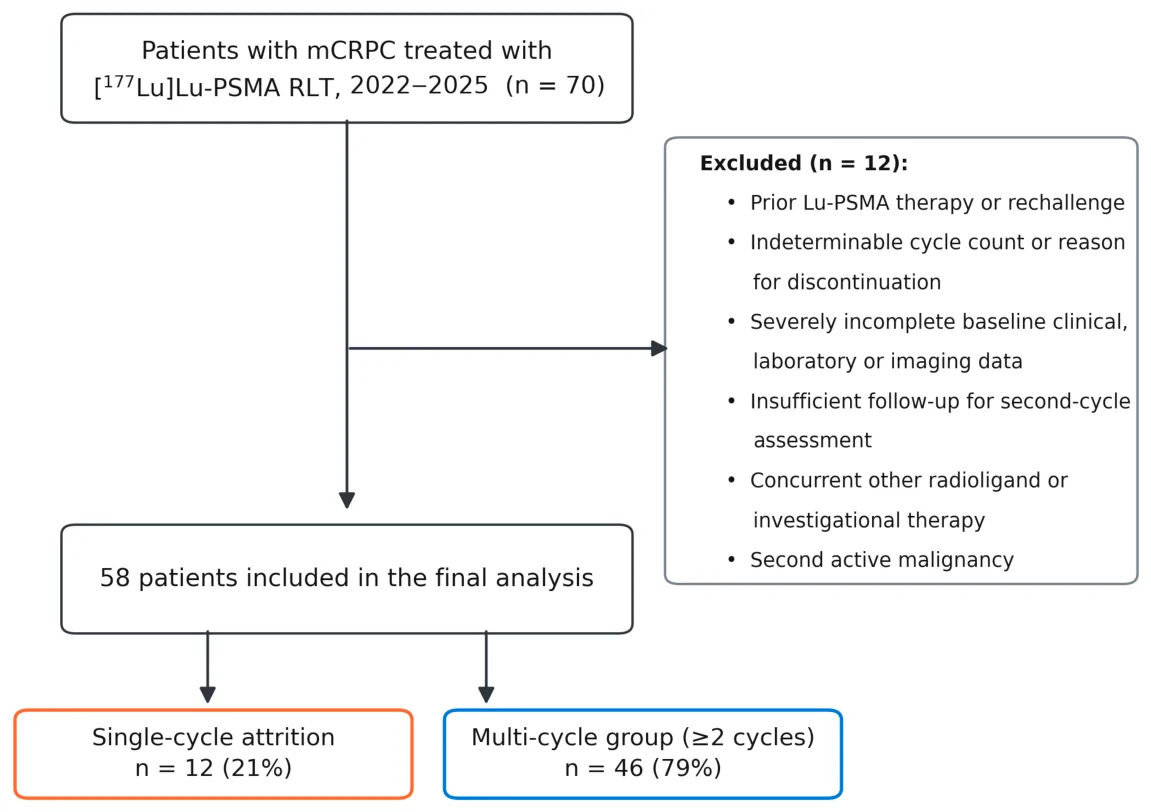
Patient flow diagram. RLT, radioligand therapy; mCRPC, metastatic castration-resistant prostate cancer; PSMA, prostate-specific membrane antigen.

Inclusion required a first cycle of [^177^Lu]Lu-PSMA-617 given for progressive mCRPC at our center within the study period and availability of the core baseline clinical and laboratory dataset. Of the 70 patients treated during the study period, 12 were excluded according to predefined criteria: prior Lu-PSMA therapy at an external center or rechallenge; indeterminable treatment-cycle count or reason for discontinuation; severely incomplete baseline clinical, laboratory or imaging data; insufficient follow-up to allow assessment of a second cycle; concurrent other radioligand therapy or investigational combination treatment; and a second active malignancy expected to affect prognosis. The remaining 58 patients constituted the analysis cohort (Figure 1).

### 2.3. Definitions and Outcome

SCA was defined as permanent non-initiation of a second RLT cycle after the first administration, established from chart review as death or rapid, irreversible clinical deterioration before the planned second cycle, or as treatment-limiting toxicity that led the multidisciplinary team to abandon further RLT. Toxicity was graded according to the Common Terminology Criteria for Adverse Events (CTCAE) version 5.0. Baseline laboratory values were those obtained within 2 weeks before the first cycle. Overall survival (OS) was measured from the date of the first cycle, and biochemical response was defined as a PSA decline of at least 50% from baseline at the week-8 assessment. Each patient was assigned a single primary reason for attrition based on the multidisciplinary-team records: death or rapid, irreversible clinical deterioration that prevented the planned second cycle, or treatment-limiting hematotoxicity that led to withholding retreatment in a patient who was otherwise able to continue. The two categories did not overlap in this cohort and were therefore mutually exclusive.

### 2.4. Candidate Baseline Predictors

Candidate predictors were specified before analysis and restricted to information available before the first administration. They comprised age and ECOG performance status; tumor grade (Gleason score and International Society of Urological Pathology [ISUP] grade group); disease burden (baseline PSA, more than 20 bone lesions, visceral metastases, nodal metastases); prior treatment (chemotherapy, radiotherapy, type of androgen-receptor pathway inhibitor); and routine pretreatment laboratory values (hemoglobin, platelets, leukocytes, lymphocytes, C-reactive protein (CRP), alkaline phosphatase (ALP), creatinine).

### 2.5. Statistical Analysis

Continuous variables were summarized as median with interquartile range (IQR) and compared with the Mann–Whitney U test; categorical variables were compared with the Fisher exact test. Benjamini–Hochberg false-discovery-rate adjustment was applied across the 18 pre-specified univariable baseline comparisons, and adjusted *q* < 0.05 was considered significant. The discrimination of individual markers was quantified by the area under the receiver operating characteristic (ROC) curve (AUC) with 2000-sample bootstrap 95% confidence intervals (CI) and Youden-index cut-offs. Markers remaining significant after adjustment were combined into an equally weighted composite score, one point per marker at its Youden cut-off. Score performance was summarized by the AUC, by exact Clopper–Pearson 95% CIs for classification measures at each threshold and by observed attrition across score strata; internal validity was assessed with an optimism-corrected bootstrap in which the entire derivation pipeline, including variable selection and cut-off derivation, was repeated in each of 1000 resamples. Overall survival was analyzed with Kaplan–Meier estimates, log-rank tests and univariable Cox regression, with median follow-up estimated by reverse Kaplan–Meier and bootstrap CIs for median survival. Multivariable modeling was not attempted given the small number of events. In a post hoc analysis, the association between the score and week-8 PSA response was examined among multi-cycle patients. All bootstrap confidence intervals were computed with the percentile method under fixed random seeds (42 for interval estimation, 7 for the optimism bootstrap), ensuring reproducibility. Statistical analyses were performed using Python 3.11.15 with the libraries SciPy 1.17.1, statsmodels 0.14.6 and scikit-learn 1.8.0.

## 3. Results

### 3.1. Baseline Characteristics

**Figure 2:**
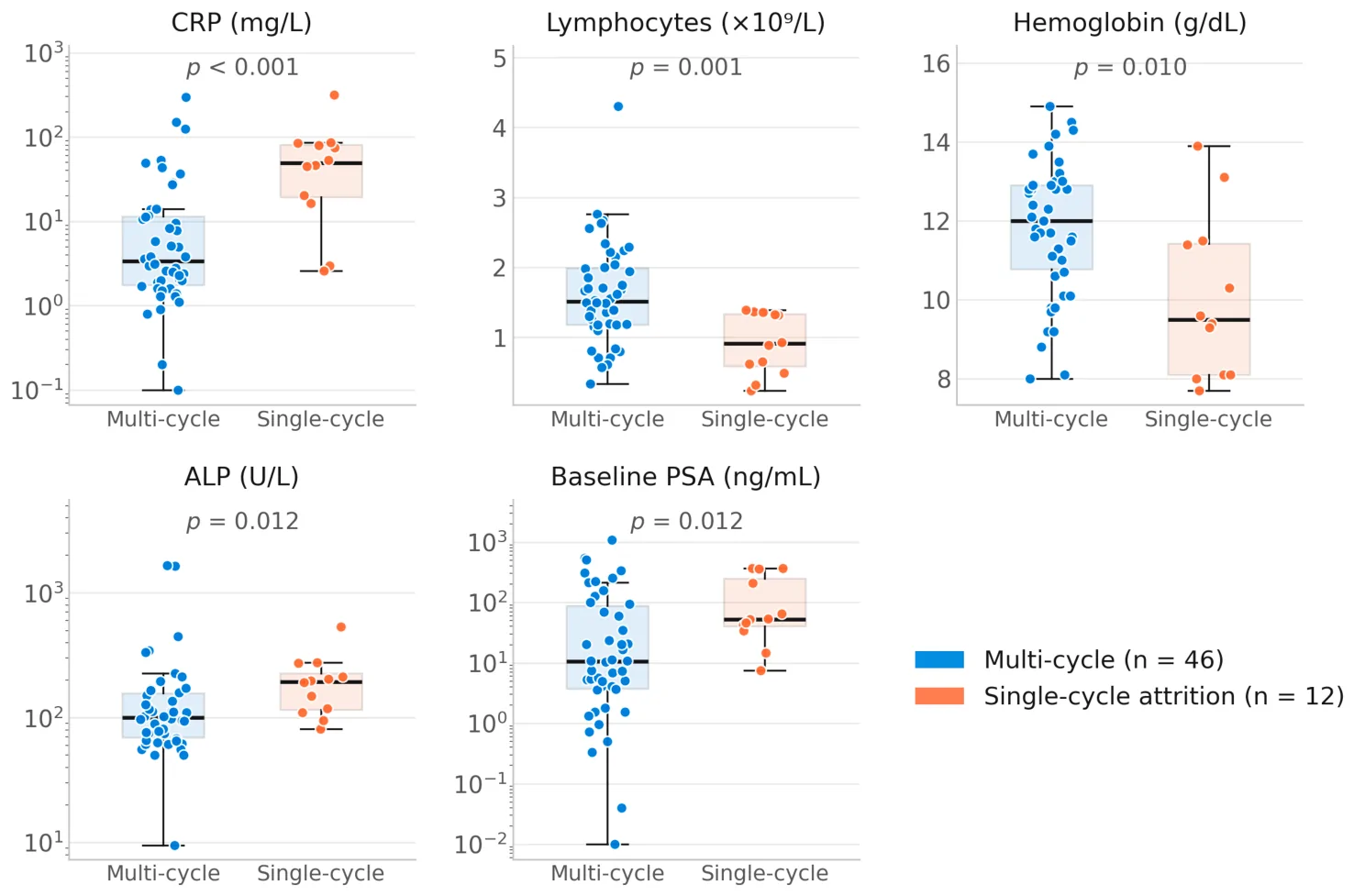
Baseline CRP, lymphocyte count, hemoglobin, ALP and PSA by treatment-exposure group (logarithmic scales where indicated; boxes: median and IQR; points: individual patients). *p* values: Mann–Whitney U test. ALP, alkaline phosphatase; CRP, C-reactive protein; PSA, prostate-specific antigen.

The 58 assessable patients had a median age of 73 years (IQR 66–78); 82% (47/57) had a Gleason score of 8–10, 63% (35/56) had received prior chemotherapy, 74% (40/54) had received prior radiotherapy and 56% (30/54) had more than 20 bone lesions (Table 1; denominators reflect the available-case analysis specified in the table footnotes). Median follow-up by reverse Kaplan–Meier was 26 months. Five routine laboratory parameters separated single-cycle from multi-cycle patients: CRP (49.5 [19.5–80.9] vs. 3.4 [1.8–11.5] mg/L, *p* < 0.001), lymphopenia (0.91 [0.59–1.34] vs. 1.52 [1.18–2.00] ×10^9^/L, *p* = 0.001), anemia (hemoglobin 9.5 [8.1–11.4] vs. 12.0 [10.8–12.9] g/dL, *p* = 0.010) and high skeletal tumor burden, reflected by ALP (194 [116–228] vs. 100 [69–157] U/L, *p* = 0.012) and baseline PSA (52.8 [40.1–247.5] vs. 10.7 [3.8–88.4] ng/mL, *p* = 0.012) (Table 2, Figure 2). All five parameters remained significant after false-discovery-rate adjustment (CRP *q* = 0.009; lymphocytes *q* = 0.012; hemoglobin, ALP and PSA *q* = 0.044). ECOG performance status was higher in single-cycle patients (median 1.5 [IQR 1–2] vs. 1 [1–1]; *p* = 0.045), although this difference did not remain significant after false-discovery-rate adjustment (*q* = 0.134); age, Gleason score, ISUP grade group, extent of bone disease, visceral or nodal metastases and prior treatments did not differ significantly between the groups.

### 3.2. Incidence and Causes of Failure to Reach a Second Cycle

**Figure 3:**
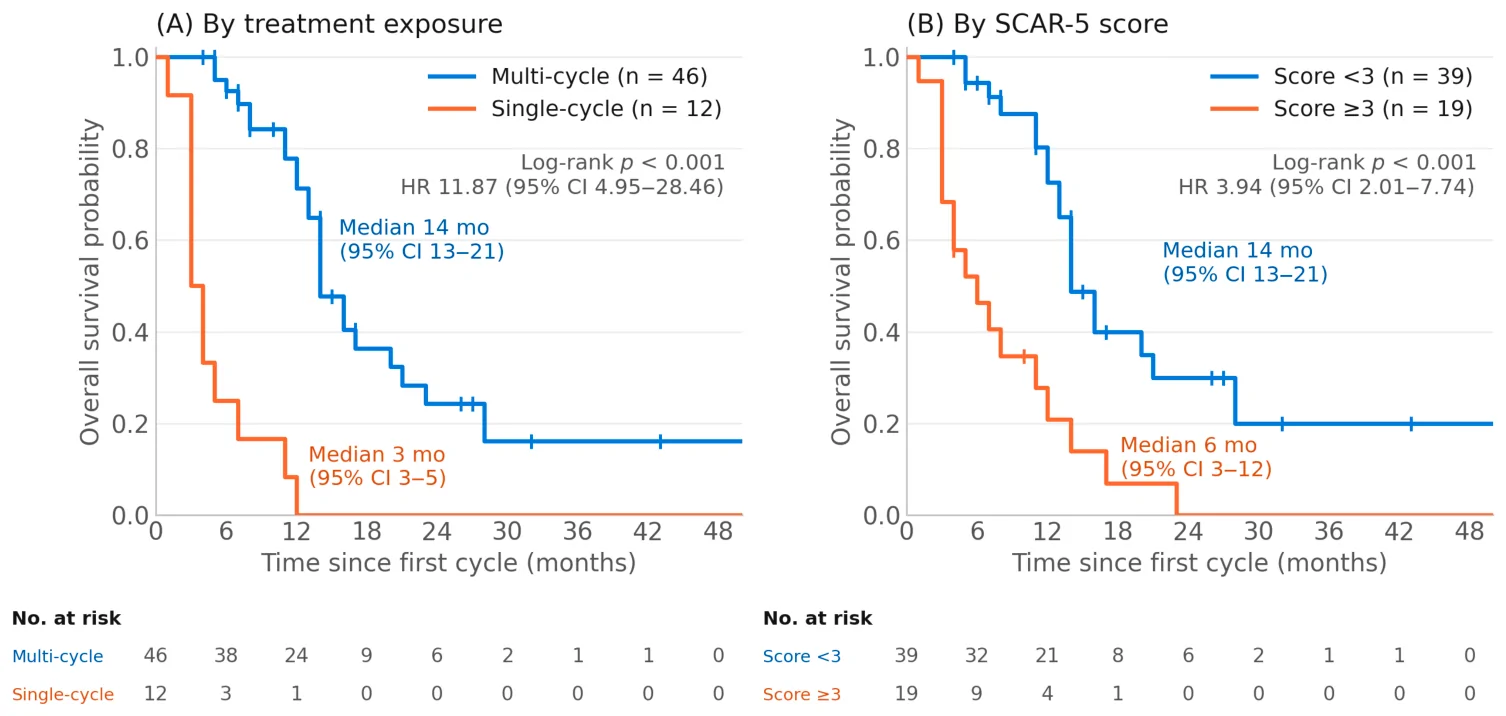
Kaplan–Meier overall survival with numbers at risk. (**A**) By treatment exposure (single-cycle vs. multi-cycle); log-rank *p* < 0.001, shown descriptively given the exposure-defined grouping. (**B**) By SCAR-5 score (≥3 vs. <3); log-rank *p* < 0.001, univariable Cox HR 3.94 (95% CI 2.01–7.74). CI, confidence interval; HR, hazard ratio; SCAR-5, five-item Single-Cycle Attrition Risk score.

Twelve of the 58 patients (20.7%) never received a second cycle. The primary reasons, classified as specified in Section 2.3, did not overlap: in 7 patients rapid, irreversible clinical deterioration precluded the planned second cycle and was followed by death, 1 patient died before the planned second cycle, and in 4 patients treatment-limiting grade 3–4 hematotoxicity (grade 4 bicytopenia in 2) led the multidisciplinary team to withhold retreatment. All 12 patients had initially been considered candidates for multi-cycle therapy. All 12 patients died, with a median OS of 3 months (95% CI 3–5) from the single administration; median OS among multi-cycle patients, who received a median of 2 cycles (range 2–7), was 14 months (95% CI 13–21; 24 of 46 deaths; Figure 3A). Notably, an early PSA decline had been documented in 5 of the 12 SCA patients, indicating that a favorable first-cycle biochemical signal did not translate into continued treatment or survival.

### 3.3. Discrimination of Individual Markers

**Figure 4:**
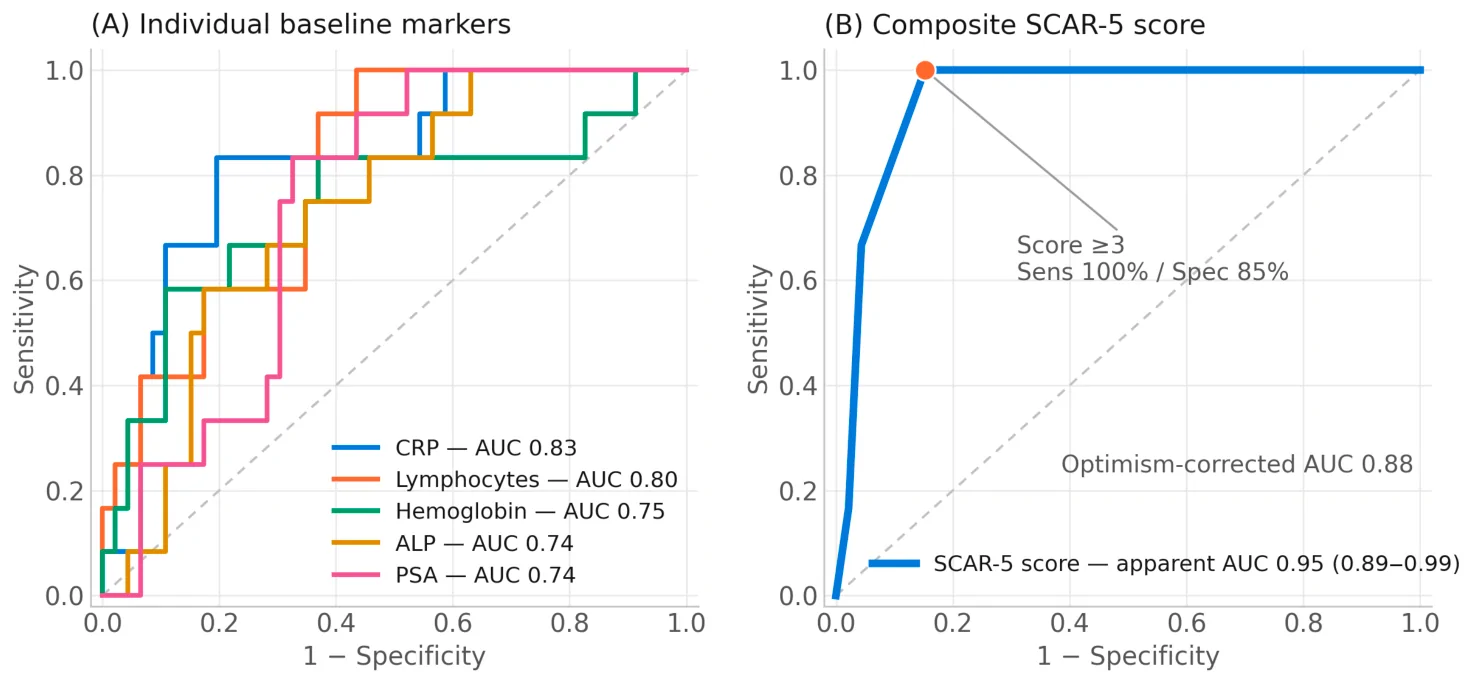
(**A**) ROC curves of the five individual baseline markers. (**B**) ROC curve of the composite SCAR-5 score; the marked operating point corresponds to a score ≥ 3. Apparent AUC is shown with the optimism-corrected estimate (0.88) from the full-pipeline bootstrap. ALP, alkaline phosphatase; AUC, area under the receiver operating characteristic curve; CRP, C-reactive protein; PSA, prostate-specific antigen; SCAR-5, five-item Single-Cycle Attrition Risk score.

Each of the five parameters discriminated SCA on its own (Table 3, Figure 4A): CRP reached an AUC of 0.83 (95% CI 0.69–0.95) and lymphocyte count an AUC of 0.80 (95% CI 0.67–0.92), while hemoglobin, ALP and baseline PSA each reached an AUC of approximately 0.74 to 0.75. No single parameter combined high sensitivity with high specificity at a clinically usable threshold (Table 3), which motivated their combination into a composite score.

### 3.4. The SCAR-5 Score

**Figure 5:**
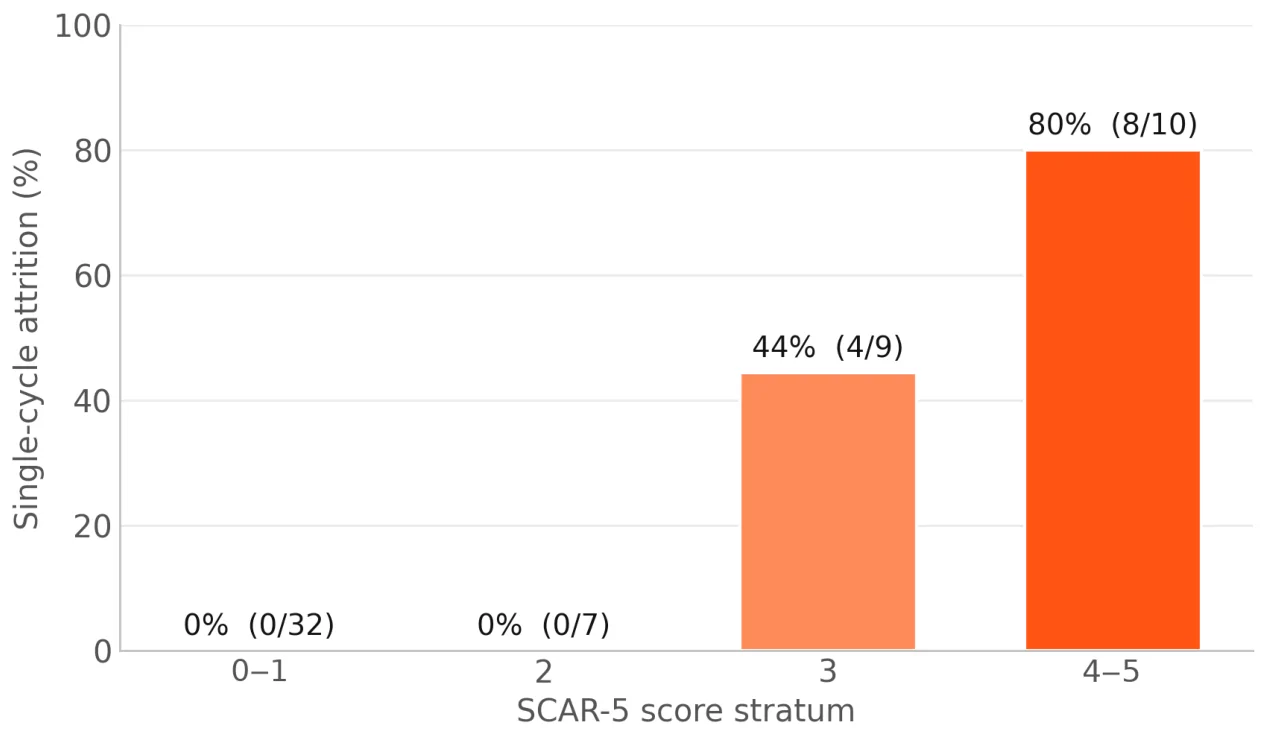
Observed single-cycle attrition across SCAR-5 strata; bar labels give the attrition percentage and events/total per stratum. SCAR-5, five-item Single-Cycle Attrition Risk score.

The five markers remaining significant after adjustment, all routine laboratory values, were combined into the five-item Single-Cycle Attrition Risk score (SCAR-5), with one point per marker at its Youden cut-off: CRP ≥ 16.5 mg/L, lymphocytes ≤ 1.39 × 10^9^/L, hemoglobin ≤ 9.6 g/dL, ALP ≥ 191 U/L and PSA ≥ 34.2 ng/mL. In the full-pipeline optimism bootstrap, the score reached an optimism-corrected AUC of 0.88 (apparent AUC 0.95, 95% CI 0.89–0.99; mean optimism 0.07; Figure 4B); given the internal derivation, the corrected estimate should be regarded as the primary measure of discrimination. Observed attrition rose steeply across score strata: 0 of 32 at scores of 0–1, 0 of 7 at a score of 2, 4 of 9 (44%) at a score of 3 and 8 of 10 (80%) at scores of 4–5; the distribution across individual score values is given in Table 4 (Figure 5). No SCA was observed below score 3 in this cohort (0 of 39 patients, two thirds of the cohort); because the exact upper 95% confidence limit of this proportion is 9.0%, a clinically relevant residual risk in low-scoring patients cannot be excluded. A threshold of ≥2 excluded SCA with sensitivity 100% (95% CI 73.5–100) and negative predictive value 100% (95% CI 89.1–100) at specificity 70%; a threshold of ≥3 identified all events at higher specificity, with sensitivity 100% (95% CI 73.5–100), specificity 85% (95% CI 71.1–93.7), positive predictive value 63% (95% CI 38.4–83.7) and negative predictive value 100% (95% CI 91–100); and a threshold of ≥4 maximized specificity (96%, 95% CI 85.2–99.5) with sensitivity 67% and a positive predictive value of 80% (Table 3 and Table 4). The score also stratified OS (Figure 3B): median OS was 6 months (95% CI 3–12) for scores ≥ 3 versus 14 months (95% CI 13–21) for scores < 3, with a log-rank *p* < 0.001 and a univariable Cox hazard ratio of 3.94 (95% CI 2.01–7.74; *p* < 0.001). Among multi-cycle patients with a documented biochemical response assessment (*n* = 39), PSA response rates did not differ significantly between scores <3 and ≥3 (53% vs. 40%; *p* = 0.66), suggesting that the score reflects the ability to continue treatment rather than the antitumor activity of RLT. To characterize the score strata beyond the laboratory components of SCAR-5, patients with scores ≥ 3 (*n* = 19) and <3 (*n* = 39) were compared with respect to their baseline clinical features (exploratory available-case comparisons). Patients with scores ≥ 3 had a poorer ECOG performance status (median 1 [IQR 1–2] vs. 1 [0–1], *p* = 0.021; ECOG ≥ 2 in 9/19 vs. 7/39, *p* = 0.029), whereas age (median 75 vs. 72 years, *p* = 0.23), Gleason score, ISUP grade group, prior treatments and the distribution of bone (>20 lesions in 71% vs. 49%, *p* = 0.15), visceral and nodal metastases did not differ significantly. Thus, apart from a poorer performance status, consistent with the frailty phenotype captured by the score, the survival separation between the strata in Figure 3B was not accompanied by differences in tumor grade, disease distribution or treatment history.

## 4. Discussion

In this consecutive real-world cohort, one in five patients who initiated Lu-PSMA RLT did not receive a second cycle. All twelve died, half of them within three months, and in five a documented early PSA decline translated into neither continued treatment nor survival. Because reaching a second cycle itself presupposes surviving until it, the survival contrast with multi-cycle patients does not measure a treatment effect; what the observed trajectories do show is that these patients spent much of their final months on a fractionated treatment they could not complete, with irradiation, repeated hospital attendance and, in one third, grade 3–4 hematotoxicity, and that current eligibility criteria did not identify them prospectively [6]. Single-cycle exposure is visible in earlier cohorts: 19% of patients in an early tertiary-center series received only one cycle [12], and early discontinuation within the first three to four cycles, with associated baseline factors including anemia and elevated ALP, has been described [13]. The present study differs from this literature in examining specifically the first-to-second-cycle transition as a pre-specified endpoint and in proposing an exploratory, internally derived laboratory index for it.

The baseline profile associated with subsequent attrition was captured by five routinely available laboratory parameters, and these map onto two complementary biological axes. The first is an axis of systemic inflammation and depleted immune–hematopoietic reserve, represented by the two strongest components: elevated CRP and lymphopenia. Systemic inflammation is an established adverse prognostic feature across malignancies [15], and the same direction has been reported for RLT specifically, with elevated CRP predicting shorter survival in an early analysis of Lu-PSMA therapy [9] and baseline hematological impairment and systemic inflammatory markers being associated with reduced treatment tolerance and inferior outcomes [16,17]. The second is an axis of skeletal tumor burden with marrow encroachment, represented by the remaining three components: anemia, elevated ALP and elevated PSA. Extensive osseous and marrow involvement is a recognized determinant of higher-grade cytopenias during RLT [18,19], and both anemia and elevated ALP likewise carried adverse prognostic weight in the same early analysis [9]; these three items quantify the burden axis through objective measurements rather than reader-dependent image interpretation. That all five components retained significance after false-discovery-rate adjustment despite the limited sample size suggests that they describe a single coherent phenotype of inflammation, catabolism and advanced disease rather than five independent signals. The contribution of the present study is the demonstration that this phenotype, summarized in a five-item score, separated a stratum with no observed attrition (scores 0–2, two thirds of the cohort) from one in which most first cycles did not lead to further treatment.

Several factors may explain why this subgroup is not identified by current selection practice. The pivotal trials were conducted under eligibility criteria that routine care does not reproduce: VISION required adequate hematological and organ reserve, and TheraP additionally employed fluorodeoxyglucose (FDG) PET screening to exclude discordant disease [1,2], whereas the populations now treated in clinical practice are older, frailer and more heavily pretreated, as registry data confirm [10]. The median exposure of two cycles and the 14-month median survival of our multi-cycle group are themselves consistent with real-world outcomes in later-line disease [10], underscoring this difference between trial and routine populations. The guideline does recognize short anticipated survival (below 3 months) and poor performance status as relative contraindications [6]; the practical difficulty is that anticipated survival is a subjective estimate, and performance status is often inconsistently documented in routine clinical records. Notably, no single-cycle patient had an ECOG status of 3 or higher, whereas five multi-cycle patients did, so the guideline threshold alone would not have identified the attrition group. In addition, existing prediction instruments address a different clinical question: the nomograms of Gafita and colleagues estimate expected overall and PSA-progression-free survival on a continuous scale [8], and single-center analyses have identified prognostic laboratory markers [9,17], but none of these tools was developed for the operational, binary question of whether a patient will be able to receive more than one cycle. Defining attrition itself as the study endpoint translates prognostic information into a criterion that is directly applicable at the time of the treatment decision, and SCAR-5 may be read as an attempt to operationalize the existing guideline caution with objective, routinely measured parameters. SCAR-5 is not intended to replace these instruments: nomogram-based survival estimates, guideline-defined relative contraindications and clinical assessment retain their roles, and the score could be integrated into existing workflows as an inexpensive first-pass flag that triggers the individualized evaluation these frameworks already require.

The intended application of such a score warrants careful delineation, and it should not serve as an automatic exclusion rule: even at scores of 4–5, one in five patients completed multi-cycle therapy, and meaningful responses have been reported in patients with extensive marrow involvement [18]. A high score should therefore not be read as evidence that treatment cannot help; it identifies patients who may still derive benefit but who are too frail, and carry too great a tumor and marrow burden, to withstand full-intensity therapy. Within this understanding, SCAR-5 may structure the individualized risk–benefit assessment already required by current guidelines [6]. Patients with scores of 0–2, two thirds of this cohort, experienced no attrition in our data; however, the exact upper 95% confidence limit of 9.0%, the small number of patients scoring exactly 2 (*n* = 7) and the absence of external validation preclude reading a low score as evidence of safety. Pending validation, a low score should be interpreted as the absence of an identified warning signal rather than as reliable protection from attrition. A score of 3 identifies patients in whom pretreatment optimization, including correction of anemia and evaluation of the inflammatory process underlying an elevated CRP, together with a planned reassessment before the second cycle, appears warranted. At scores of 4–5, four of five first cycles were not followed by further treatment; these scores should prompt a structured multidisciplinary discussion of treatment goals, weighing immediate RLT against best supportive care, alternative systemic options or short-interval reassessment after optimization. Because treatment-limiting hematotoxicity occurred in one third of SCA patients and treatment intent is not curative, dose de-escalation is a rational strategy for precisely this group: a recent prospective phase II study reported preserved efficacy with favorable tolerability at 5.5 GBq per cycle [20]. In this context, our hypothesis-generating results may help identify candidates for dose-de-escalated or otherwise individualized treatment strategies once external and ideally prospective validation is available.

The implications of this decision extend beyond toxicity. For patients whose expected survival is measured in weeks to months, each treatment cycle absorbs a considerable proportion of the remaining lifetime in healthcare-related activity, including travel, admission, radiation-protection isolation and recovery, a burden increasingly conceptualized in oncology as time toxicity. Administrations that do not lead to a completed treatment course also have implications for resource allocation, as radioligand treatment capacity, isotope supply and shielded inpatient facilities are limited in most healthcare systems, and the cost-effectiveness of Lu-PSMA RLT itself appears contingent on the survival benefit actually realized, and therefore on patient selection [14]. Furthermore, aggressive tumor-directed therapy during the final weeks of life is an established indicator of low-quality end-of-life care. A validated attrition score could provide a quantitative instrument for aligning the delivery of RLT with these broader goals of care.

The observation that five of the twelve SCA patients exhibited an early PSA decline warrants consideration. A favorable biochemical signal after the first cycle translated into neither continued treatment nor survival, presumably because death and hematological failure in these patients were determined by overall disease burden and physiological exhaustion rather than by inadequate targeting of the PSMA pathway. This interpretation is supported by the absence of an association between the score and PSA response among multi-cycle patients in our cohort, and it reinforces the central design principle of the present study: a pretreatment selection framework for RLT should be anchored in baseline physiology rather than in early response markers, which may be discordant with the overall clinical trajectory.

This study has limitations. Its retrospective, single-center design and the small number of events limit statistical power, precluded multivariable modeling and leave wide confidence intervals around all estimates. Beyond limited power, this design carries specific risks of bias: selection bias arising from tertiary referral patterns, ascertainment bias from chart-based data collection, and center-specific continuation practice that may not generalize to other institutions. In addition, several baseline variables remained incompletely documented (nodal status in six patients; radiotherapy history, bone-lesion count and visceral status in four each; chemotherapy history in two; Gleason score in one), so the corresponding comparisons are available-case analyses. SCAR-5 was derived and evaluated in the same cohort, and although the optimism-corrected bootstrap addresses this internally, the score remains exploratory until it is validated externally. Quantitative baseline PSMA-PET biomarkers were not analyzed: mean standardized uptake value (SUVmean), PSMA-positive tumor volume and total lesion PSMA have been associated with response and survival after Lu-PSMA therapy [21], and PET-derived measures of bone-disease burden with hematologic toxicity and treatment discontinuation [19], so such parameters might add discriminatory information to the purely laboratory-based SCAR-5, which we restricted to routine laboratory values because they are objective, inexpensive and available in any treatment setting without dedicated quantification software.

## 5. Conclusions

In this real-world cohort, one in five evaluable patients initiating Lu-PSMA RLT did not proceed beyond the first treatment cycle, and this event carried a uniformly poor prognosis: all affected patients died during follow-up, half within three months. Five routinely available pretreatment laboratory parameters (CRP, lymphocyte count, hemoglobin, ALP and PSA) were associated with early treatment attrition, and the exploratory SCAR-5 score derived from them separated clearly distinct strata of treatment continuation: no single-cycle attrition was observed in the two thirds of the cohort scoring below 3 (upper 95% confidence limit 9.0%), whereas most patients scoring 4 or 5 never received a second cycle. These findings suggest that meeting current eligibility criteria does not by itself ensure that the intended multi-cycle course can be delivered.

SCAR-5 remains exploratory and should not be interpreted as an eligibility or exclusion tool, nor as a basis for individual treatment decisions before external validation; in particular, a low score should not be read as a guarantee that treatment will be completed. Subject to external and prospective validation, it may complement clinical judgment at the time of the first cycle by prompting closer reassessment, optimization of potentially reversible factors and individualized risk–benefit evaluation where scores are high. Whether dose modification or alternative treatment strategies can improve cycle completion or clinical outcomes in these high-risk patients requires prospective investigation.

## Figures and Tables

**Table 1:** Baseline clinical characteristics.

Characteristic	Single-Cycle (*n* = 12)	Multi-Cycle (*n* = 46)	*p*	*q*
Age, years, median (IQR)	76 (70–80)	71 (66–77)	0.146	0.376
ECOG performance status, median (IQR)	1.5 (1–2)	1 (1–1)	0.045	0.134
Gleason score, median (IQR) *	9 (8–9)	8 (8–9)	0.560	0.775
Gleason score 8–10 (ISUP 4–5), *n*/*N* (%) *	9/12 (75%)	38/45 (84%)	0.424	0.725
ISUP grade group, median (IQR) *	5 (4–5)	4 (4–5)	0.717	0.860
Prior chemotherapy, *n*/*N* (%) *	7/11 (64%)	28/45 (62%)	1.000	1.000
Prior radiotherapy, *n*/*N* (%) *	10/12 (83%)	30/42 (71%)	0.710	0.860
Prior ARPI (enzalutamide), *n*/*N* (%) †	6/9 (67%)	20/36 (56%)	0.712	—
Bone metastases > 20 lesions, *n*/*N* (%) *	7/10 (70%)	23/44 (52%)	0.483	0.725
Visceral metastases, *n*/*N* (%) *	3/10 (30%)	13/44 (30%)	1.000	1.000
Lymph node metastases, *n*/*N* (%) *	7/9 (78%)	26/43 (60%)	0.458	0.725

Values are median (IQR) or *n*/*N* (%). *p*: Mann–Whitney U or Fisher exact test; *q*: Benjamini–Hochberg false-discovery-rate-adjusted *p* value across the pre-specified family of 18 univariable comparisons. * Available-case analysis; denominators reflect patients with available data (Gleason score unavailable in 1 patient; prior chemotherapy in 2; prior radiotherapy, bone-lesion count and visceral status in 4 each; nodal status in 6). † Proportion receiving enzalutamide among patients treated with an androgen-receptor pathway inhibitor; *q* not applicable. ARPI, androgen-receptor pathway inhibitor; ECOG, Eastern Cooperative Oncology Group; IQR, interquartile range; ISUP, International Society of Urological Pathology.

**Table 2:** Baseline laboratory values.

Parameter (Median [IQR])	Single-Cycle (*n* = 12)	Multi-Cycle (*n* = 46)	*p*	*q*
CRP, mg/L	49.5 [19.5–80.9]	3.4 [1.8–11.5]	<0.001	0.009
Lymphocytes, ×10^9^/L	0.91 [0.59–1.34]	1.52 [1.18–2.00]	0.001	0.012
Hemoglobin, g/dL	9.5 [8.1–11.4]	12.0 [10.8–12.9]	0.010	0.044
ALP, U/L	194 [116–228]	100 [69–157]	0.012	0.044
Baseline PSA, ng/mL	52.8 [40.1–247.5]	10.7 [3.8–88.4]	0.012	0.044
Leukocytes, ×10^9^/L	5.8 [4.5–7.1]	6.9 [5.5–7.9]	0.202	0.410
Platelets, ×10^9^/L	181 [146–304]	252 [212–278]	0.205	0.410
Creatinine, mg/dL	0.86 [0.69–0.97]	0.88 [0.69–1.02]	0.816	0.918

Mann–Whitney U tests. *q*: Benjamini–Hochberg false-discovery-rate-adjusted *p* value across the 18-test family. All values were obtained within 2 weeks before the first cycle and were available for all 58 patients, except creatinine (*n* = 57). ALP, alkaline phosphatase; CRP, C-reactive protein; IQR, interquartile range; PSA, prostate-specific antigen.

**Table 3:** Discrimination of candidate baseline markers and of the SCAR-5 score.

Marker	AUC (95% CI)	Cut-off	Sensitivity	Specificity
CRP, mg/L	0.83 (0.69–0.95)	≥16.5	83%	80%
Lymphocytes, ×10^9^/L	0.80 (0.67–0.92)	≤1.39	100%	57%
Hemoglobin, g/dL	0.75 (0.55–0.91)	≤9.6	58%	89%
ALP, U/L	0.74 (0.58–0.87)	≥191	58%	83%
Baseline PSA, ng/mL	0.74 (0.60–0.86)	≥34.2	83%	67%
SCAR-5 composite score	0.95 (0.89–0.99)	≥3	100%	85%

Bootstrap (2000 resamples) 95% CIs; marker cut-offs by Youden index. SCAR-5 assigns one point per marker at the cut-off shown. Apparent (resubstitution) performance; the optimism-corrected AUC of 0.88 from the full-pipeline bootstrap constitutes the primary estimate of score discrimination. ALP, alkaline phosphatase; AUC, area under the receiver operating characteristic curve; CI, confidence interval; CRP, C-reactive protein; PSA, prostate-specific antigen; SCAR-5, five-item Single-Cycle Attrition Risk score.

**Table 4:** Distribution of SCAR-5 scores and observed single-cycle attrition (*n* = 58).

SCAR-5 Score	Patients, *n*	SCA Events, *n* (%)
0	13	0 (0%)
1	19	0 (0%)
2	7	0 (0%)
3	9	4 (44%)
4	7	6 (86%)
5	3	2 (67%)
Total	58	12 (20.7%)

SCA, single-cycle attrition; SCAR-5, five-item Single-Cycle Attrition Risk score. Percentages are per score value; grouped strata are shown in Figure 5.

## Data Availability

The data presented in this study are not publicly available because they contain confidential patient information. De-identified data may be provided by the corresponding author upon reasonable request and subject to approval by the relevant institutional ethics committee.

## Abbreviations

The following abbreviations are used in this manuscript:

ALP	Alkaline phosphatase
ARPI	Androgen-receptor pathway inhibitor
AUC	Area under the receiver operating characteristic curve
CI	Confidence interval
CRP	C-reactive protein
CT	Computed tomography
CTCAE	Common Terminology Criteria for Adverse Events
EANM	European Association of Nuclear Medicine
ECOG	Eastern Cooperative Oncology Group
FDG	Fluorodeoxyglucose
HR	Hazard ratio
IQR	Interquartile range
ISUP	International Society of Urological Pathology
mCRPC	Metastatic castration-resistant prostate cancer
OS	Overall survival
PET	Positron emission tomography
PSA	Prostate-specific antigen
PSMA	Prostate-specific membrane antigen
RLT	Radioligand therapy
ROC	Receiver operating characteristic
SCA	Single-cycle attrition
SCAR-5	Five-item Single-Cycle Attrition Risk score
SNMMI	Society of Nuclear Medicine and Molecular Imaging
SUV	Standardized uptake value
